# Laboratory features of severe vs. non-severe COVID-19 patients in Asian populations: a systematic review and meta-analysis

**DOI:** 10.1186/s40001-020-00432-3

**Published:** 2020-08-03

**Authors:** Sulmaz Ghahramani, Reza Tabrizi, Kamran B. Lankarani, Seyyed Mohammad Amin Kashani, Shahla Rezaei, Nazanin Zeidi, Maryam Akbari, Seyed Taghi Heydari, Hamed Akbari, Peyman Nowrouzi-Sohrabi, Fariba Ahmadizar

**Affiliations:** 1 Health Policy Research Center, Institute of Health, Shiraz University of Medical Sciences, Shiraz, Iran; 2 Student Research Committee, Shiraz University of Medical Sciences, Shiraz, Iran; 3 Department of Clinical Nutrition, School of Health and Nutrition, Shiraz University of Medical Sciences, Shiraz, Iran; 4 Department of Biochemistry, School of Medicine, Kerman University of Medical Sciences, Kerman, Iran; 5 Department of Biochemistry, School of Medicine, Shiraz University of Medical Sciences, Building No. 3, Zand Avenue, Shiraz, Iran; 6 Department of Epidemiology, Erasmus University Medical Center, Rotterdam, The Netherlands

**Keywords:** Laboratory features, COVID-19, Meta-analysis

## Abstract

**Background:**

More severe cases of COVID- 19 are more likely to be hospitalized and around one-fifth, needing ICU admission. Understanding the common laboratory features of COVID-19 in more severe cases versus non-severe patients could be quite useful for clinicians and might help to predict the model of disease progression. This systematic review and meta-analysis aimed to compare the laboratory test findings in severe vs. non-severe confirmed infected cases of COVID-19.

**Methods:**

Electronic databases were systematically searched in PubMed, EMBASE, Scopus, Web of Science, and Google Scholar from the beginning of 2019 to 3rd of March 2020. Heterogeneity across included studies was determined using Cochrane’s Q test and the *I*^2^ statistic. We used the fixed or random-effect models to pool the weighted mean differences (WMDs) or standardized mean differences and 95% confidence intervals (CIs).

**Findings:**

Out of a total of 3009 citations, 17 articles (22 studies, 21 from China and one study from Singapore) with 3396 ranging from 12 to1099 patients were included. Our meta-analyses showed a significant decrease in lymphocyte, monocyte, and eosinophil, hemoglobin, platelet, albumin, serum sodium, lymphocyte to C-reactive protein ratio (LCR), leukocyte to C-reactive protein ratio (LeCR), leukocyte to IL-6 ratio (LeIR), and an increase in the neutrophil, alanine aminotransferase (ALT), aspartate aminotransferase (AST), total bilirubin, blood urea nitrogen (BUN), creatinine (Cr), erythrocyte Sedimentation Rate (ESR), C-reactive protein (CRP), Procalcitonin (PCT), lactate dehydrogenase (LDH), fibrinogen, prothrombin time (PT), D-dimer, glucose level, and neutrophil to lymphocyte ratio (NLR) in the severe group compared with the non-severe group. No significant changes in white blood cells (WBC), Creatine Kinase (CK), troponin I, myoglobin, IL-6 and K between the two groups were observed.

**Interpretation:**

This meta-analysis provides evidence for the differentiation of severe cases of COVID-19 based on laboratory test results at the time of ICU admission. Future well-methodologically designed studies from other populations are strongly recommended.

## Background

The coronavirus disease 2019 (COVID-19) outbreak started in December 2019 in China has spread sharply all over the world. Reports showed that more than 212 countries and territories around the world are affected by the COVID-19 pandemic as of May 13th [1]. There have been more than 4000,000 confirmed reported COVID-19 cases affected by and more than 280,000 reported deaths until May 13th [1]. While there are parallels between COVID-19 and the severe acute respiratory syndrome (SARS), variations in the clinical characteristics of the diseases caused by the two viruses have been noted [2]. Urgent identification of clinical laboratory predictors of disease progression toward severe/critical form is an urgent necessity for clinicians to be able to stratify risks, distinguish and differentiate severe patients from the mild/moderate form of COVID-19.

Based on the clinical symptoms and laboratory test results, patients are categorized as mild, moderate, severe, and critical types [2, 3]. Mild/moderate cases include most of the affected patients (81%). Although severe and critical ones comprise only 14% and 5% of infected cases, respectively [4] they mainly need hospitalization. Almost 20% of hospitalized patients need intensive care unit (ICU) [5]. As such, the mortality rate of ICU admitted COVID-19 patients is reported quite high, nearly 61.5% die due to many different reasons [6].

Apart from the clinical symptoms and pulmonary computed tomography (CT) findings, a large number of COVID-19 confirmed patients showed laboratory fluctuations including complete blood count (CBC) variables, cardiac and coagulation parameters, renal and liver function tests, and inflammation-related factors [7, 8].

Recently, combinations of some laboratory tests have been used in some settings to show the hyperinflammation state and prognosis. These combinations include neutrophil to lymphocyte ratio (NLR) and lymphocyte to C-reactive protein ratio (LCR) [9–11].

Among the CBC parameters of COVID-19 confirmed cases, decreased lymphocytes and normal or increased monocytes have pointed out previously [7]. Combined evidence so far appears inconsistent [6, 7, 12–15]. In the previous meta-analysis, the most prevalent laboratory features that were decreased in all confirmed cases consist of albumin, high CRP, high lactate dehydrogenase (LDH), lymphopenia, and high ESR, while increase in bilirubin, cytokines, and leukocytes was less frequent [5]. Since the pandemic outbreak of COVID-19, a vast number of studies investigated the laboratory changes in confirmed COVID-19 patients, leaving the association between the routine laboratory features and the disease severity as less attention subject. Therefore, in this study, we conducted a systematic review and meta-analysis to quantify the results of previously published studies, comparing the CBC indices cardiac and coagulation parameters, electrolyte factors, renal and liver function tests, inflammation-related factors, and some new combined inflammatory laboratory tests in severe/critical versus non-severe confirmed infected cases of COVID-19.

## Methods

The Preferred Reporting Items for Systematic Reviews and Meta-analyses (PRISMA) guidelines were used for performing and reporting our systematic review and meta-analysis.

### Search strategy

Electronic databases were systematically searched in PubMed, EMBASE, Scopus, Web of Science, and Google Scholar from the beginning of 2019 to 3rd of March 2020. The reference lists of relevant studies and previous reviews manually were checked to retrieve more studies. Search terms included “2019 novel coronavirus infection” OR “COVID-19” OR “COVID19” OR “coronavirus disease 2019” OR “coronavirus disease-19” OR “2019-nCoV disease” OR “2019 novel coronavirus disease” OR “2019-nCoV infection” OR “2019-nCoV” OR “2019 novel coronavirus” OR “2019 coronavirus” OR “novel coronavirus” OR (2019 AND coronavirus). Additional file 1: Appendix 1 provides of search strategy from Scopus database.

### Inclusion and exclusion criteria

Two independent researches (M.A-K and N.Z) assessed all retrieved reports using our inclusion and exclusion criteria. Differences were resolved through consensus or discussion with a third author (R.T). Studies that met the following criteria were included in our study: original studies with cross-sectional, case–control, and cohort design in English language; studies that investigated laboratory features [includes CBC (neutrophil, lymphocyte, monocyte, eosinophil, hemoglobin, and platelet), liver and kidney functions [alanine aminotransferase (ALT), aspartate aminotransferase (AST), albumin, total bilirubin (TBIL), blood urea nitrogen (BUN), and creatinine (Cr)], myocardial enzymes [creatine kinase (CK), troponin I, and myoglobin], inflammatory factors [ESR, CRP, LDH, procalcitonin (PCT), and IL-6] serum electrolytes (sodium, potassium), coagulation functions [fibrinogen, prothrombin time (PT), and D-dimer], and glucose level; we also combined inflammatory markers including NLR, LCR, leukocyte to C-reactive protein ratio (LeCR), and leukocyte to IL-6 ratio (LeIR). For a variable to be included in analysis, at least three studies that reported or appropriated to calculate the mean changes (standard deviation (SD)) of intended laboratory features in severe vs. non-severe COVID-19 were needed. Severe cases were defined based on severe cases of America thoracic society [16] or interim guidance of World Health Organization definition [17] or severe/critical case based on China’s National Health Commission definition [18] or Acute Respiratory Distress Syndrome (ARDS) based on Berlin definition [19] or admission to ICU. Otherwise, patients were defined as non-severe. We excluded studies that were review, case report, case series, letter to editor, and abstracts without full text.

### Data extraction

Two researchers (SMAK and NZ) were independently extracted the following data from the included studies: first author’s name, year of publication, type of publication, country, patient characteristics, total sample size, number of patients in severe and non-severe groups, mean (SD) of laboratory parameters in severe and non-severe groups. Differences between the two researchers were resolved by consensus or discussion with a third author (PN-S).

### Quality assessment

The quality of the included articles was critically assessed using the Newcastle–Ottawa Scale (NOS). This tool was conducted according to three aspects including selection, comparability, and exposure/outcome. A study with an NOS score of ≥ 7 was considered as good quality. Table 1 shows the results of the quality assessment of included studies based on NOS.

**Table 1:** Characteristics of included studies

Authors	Publication year	Country	Sample size (severe/non-severe)	Study design^a^	Patients (severe/non-severe)	Age group (severe vs non-severe)	Quality assessment (score)	References
Cai et al.	2020	China	58/240	Cross-sectional	Severe/non-severe	64 ± 7.41, 40 ± 18.53	8	[12]
Cao (Min) et al.	2020	China	19/179	Single-center cohort	ICU/Non-ICU	63.7 ± 16.8, 48.6 ± 15.6	7	[13]
Cao (Weiliang) et al.	2020	China	21/107	Retrospective study	Severe/non-severe	NR	4	[14]
Deng (a) et al.	2020	China	59/10	Cross-sectional	Severe/ordinary	61.4 ± 16.7 (all patients)	4	[7]
Deng (b) et al.	2020	China	36/9	Cross-sectional	ICU/ordinary cases	61.4 ± 16.7 (all patients)	4	[7]
Guan et al.	2020	China	173/926	Cohort	Severe/non-severe	52 ± 18.53, 45 ± 17.05	6	[20]
Huang et al.	2020	China	13/28	Cohort	ICU/Non-ICU	49 ± 14.82, 49 ± 12.23	5	[7]
Jian-ya et al.	2020	China	7/44	Retrospective, single-center case series	Severe/non-severe	52 ± 11.86, 44 ± 11.86	3	[21]
Li et al.	2020	China	25/58	Retrospective study	Severe or critical/ordinary group	53.7 ± 12.3, 41.9 ± 10.6	5	[25]
Liu (Jingyuan) et al.	2020	China	17/44	Prospective single-center study	Severe or critical/common type	56 ± 9.75, 41 ± 18.75	5	[23]
Liu (Songqiao) (a) et al.	2020	China	53/97	Retrospective multicenter cohort study	Severe or critical/asymptomatic or Mild	60.09 ± 13.86, 35.96 ± 19.88	9	[20]
Liu(Songqiao) (b) et al.	2020	China	27/470	Retrospective multicenter cohort study	Severe or critical/moderate	60.09 ± 13.86, 44.47 ± 15.62	9	[20]
Liu (Yanli) et al.	2020	China	26/56	Retrospective study	ARDS/Non-ARDS	61 ± 13.34, 49 ± 13.34	8	[24]
Wang et al.	2020	China	36/102	Retrospective, single-center case series (cohort)	ICU/Non-ICU	66 ± 15.57, 51 ± 18.53	7	[25]
Young et al.	2020	Singapore	6/12	Descriptive case series	Required supplemental O2/Did not require supplemental O2	56 ± 6.5, 37 ± 6.25	6	[27]
Zhang (Fengqin) (a) et al.	2020	China	9/23	Retrospective single center	Critical/common	50.3 ± 14, 40.8 ± 12.2	6	[23]
Zhang (Fengqin) (b) et al.	2020	China	26/23	Retrospective single center	Severe/common	48.9 ± 13.5, 40.8 ± 12.2	6	[23]
Zhang (Jin-jin) et al.	2020	China	58/82	Cross-sectional	Severe/non-severe patients	64 ± 15.5, 51.5 ± 13	6	[22]
Zheng (a) et al.	2020	China	3/71	Cross-sectional	Severe or critical)/non-severe	67.875 ± 12.22, 44.845 ± 16.79	5	[15]
Zheng (b) et al.	2020	China	3/9	Cross-sectional	Severe or critical/Mild	67.875 ± 12.22, 39.444 ± 14.32	5	[15]
Zheng (c) et al.	2020	China	2/15	Cross-sectional	Severe and critical/asymptomatic infected	67.875 ± 12.22, 32.667 ± 22.68	5	[15]
Zhu et al.	2020	China	43/71	Retrospective cohort study	Severe group/non-severe group	76 ± 6.67, 77 ± 8.15	8	[25]

^a^ study design whether stated clearly in methods or perceived from methods

### Statistical analysis

All statistical analyses were performed using STATA version 12.0 (Stata Corp., College Station, TX). Heterogeneity among included studies was assessed using Cochrane’s Q test or the *I*^2^ statistic. *I*^2^ above 70% and Cochrane’s Q test with *P* < 0.05 were considered as the existence of significant heterogeneity.

Laboratory factors were estimated as the mean (SD) difference with 95% confidence intervals (CIs) between severe and non-severe groups. Weighted mean difference (WMD) with the random-effects model (DerSimonian–Laird method) was used to pool the mean differences of each laboratory factors in studies with the same clinical units and measures; otherwise, the standardized mean difference (SMD) was applied. We used a series of sensitivity analysis to assess the robustness of our findings; applying the leave-one-out method to test the impact of each included study on the pooled WMDs or SMDs. The potential evidence of publication bias was assessed using the Egger regression and Begg’s rank correlation tests.

## Results

**Fig. 1:**
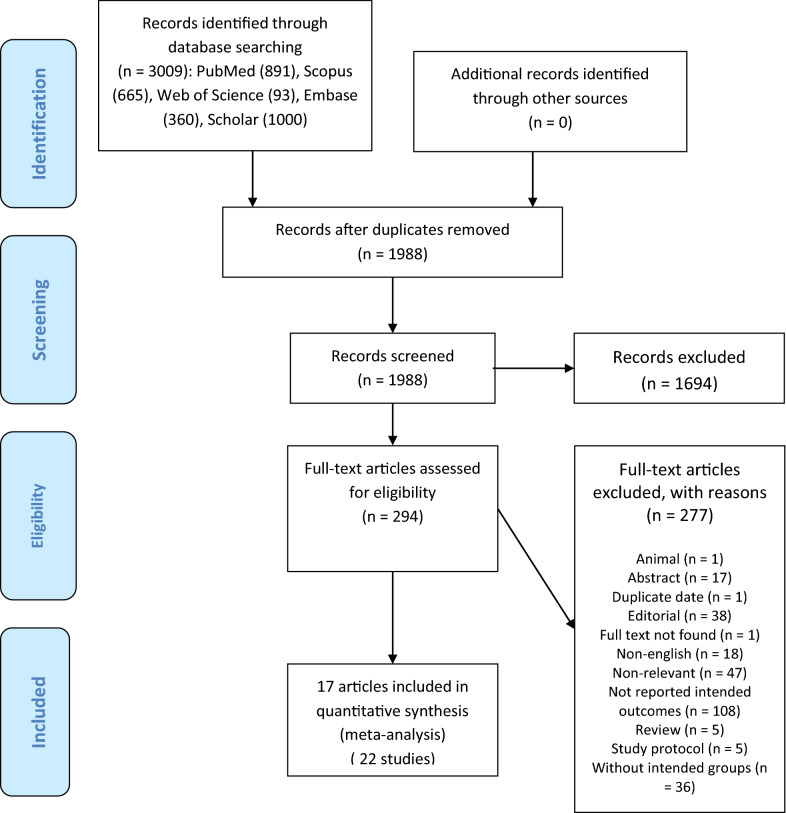
The process of study identification and selection

A total of 3009 citations were identified through electronic database searches. Of these, 1021 were duplicate reports. After screening titles and abstracts, 1694 articles were excluded and 294 full-text articles were retrieved for more assessment. Finally, 17 articles (or 22 studies) were found to be eligible for this meta-analysis. We summarized the process of study identification and selection in Fig. 1.

All included articles were conducted in China [7, 12–15, 20–26] except one that was performed in Singapore [27]. Twenty-three studies of 17 articles included 3396 (ranging from 12 to 1099) patients who were 720 in severe and 2676 in non-severe groups. The number of studies with different laboratory tests is as following: 22 studies were on lymphocyte, 21 on WBC, 18 on neutrophil and CRP, 17 on platelet, 15 on ALT, 14 on AST, Cr, and CK, 12 on albumin, PCT, and D-dimer, 11 on LDH, 10 on monocyte, 9 on hemoglobin, TBIL, BUN, ESR, sodium, and potassium, 8 on PT, 6 on IL-6, 5 on eosinophil and troponin I, 4 on fibrinogen and glucose, and 3 on myoglobin. Most studies clearly stated that data were basic (on admission/before treatment) laboratory test results [7, 12–14, 20–25, 27]. The study characteristics of these 23 included articles are presented in Table 1.

### Main outcomes

The mean difference forest plots of laboratory features in severe vs. non-severe hospitalized patients with COVID-19 are shown in Additional file 2: Appendix 2: 2a– h.

### CBC with differential counts of WBC

Using random-effects model, our meta-analyses showed a significant decrease in the WMD of lymphocyte [WMD = − 0.43 × 10^9^ per L; 95% CI − 0.56, − 0.30, *P* < 0.001; *I*^2^ = 90.1% (with 22 studies)], monocyte [WMD = − 0.06 × 10^9^ per L; 95% CI − 0.12, − 0.01, *P* = 0.032; *I*^2^ = 71.8% (with 10 studies)], and eosinophil [WMD = − 0.03 × 10^9^ per L; 95% CI − 0.05, − 0.00, *P* = 0.037; *I*^2^ = 86.1% (with 5 studies)], hemoglobin [WMD = − 5.94 g/L; 95% CI − 8.23, − 3.64, *P* < 0.001; *I*^2^ = 0.0% (with 9 studies)], platelet [WMD = − 27.97 × 10^9^ per L; 95% CI − 39.60, − 16.35, *P* < 0.001; *I*^2^ = 55.8% (with 17 studies)], and increased in the WMD of neutrophil [WMD = 0.74 × 10^9^ per L; 95% CI 0.16, 1.33, *P* = 0.013; *I*^2^ = 74.6% (with 18 studies)], in the severe group compared with the non-severe group. However, no significant differences were found in WBC [WMD = 0.55 × 10^9^ per L; 95% CI − 0.09, 1.19, *P* = 0.094; *I*^2^ = 81.3% (with 21 studies)] between the two groups were observed (Additional file 2: Appendix 2a, Fig: A–G).

### Laboratory tests for liver and kidney function

The results indicated a significant decrease in the WMD of albumin [WMD = − 4.20 g/L; 95% CI − 5.99, − 2.41, *P* < 0.001; *I*^2^ = 73.9% (with 12 studies)], and increased in the WMD of ALT [WMD = 6.65 U/L; 95% CI 4.21, 9.09, *P* < 0.001; *I*^2^ = 0.0% (with 15 RCTs)], AST [WMD = 11.91 U/L; 95% CI 8.29, 15.53, *P* < 0.001; *I*^2^ = 46.1% (with 14 studies)], TBIL [WMD = 0.08 mg/dL; 95% CI 0.03, 0.14, *P* = 0.005; *I*^2^ = 0.0% (with 9 studies)], BUN [WMD = 2.34 mg/dL; 95% CI 0.66, 4.03, *P* = 0.006; *I*^2^ = 39.3% (with 9 studies)], and Cr [WMD = 0.08 mg/dL; 95% CI 0.03, 0.12, *P* < 0.001; *I*^2^ = 0.0% (with 14 studies)] in the severe group compared with the non-severe group (Additional file 2: Appendix 2b, Fig: A–F).

### Myocardial enzymes and myoglobin

The pooled findings showed no significant differences between the two groups of COVID-19 patients on myocardial enzymes and myoglobin, including CK [WMD = − 3.01 U/L; 95% CI − 12.91, 6.90, *P* = 552; *I*^2^ = 51.7% (with 14 studies)], troponin I [SMD = 0.27; 95% CI − 0.14, 0.67, *P* = 0.193; *I*^2^ = 77.3% (with 5 studies)], and myoglobin [WMD = 8.11 ng/mL; 95% CI − 6.10, 22.33, *P* = 0.263; *I*^2^ = 73.2% (with 3 studies)] (Additional file 2: Appendix 2c, Fig: A–C).

### Inflammatory markers

Our findings of inflammatory markers showed a significant increase in the WMD of ESR [WMD = 27.67 mm/h; 95% CI 22.94, 32.40, *P* < 0.001; *I*^2^ = 22.3% (with 9 studies)], CRP [WMD = 36.61 mg/L; 95% CI 24.40, 48.82, *P* < 0.001; *I*^2^ = 91.9% (with 18 studies)], LDH [WMD = 102.15 U/L; 95% CI 72.76, 131.53, *P* < 0.001; *I*^2^ = 50.3% (with 11 studies)], and PCT [WMD = 0.03 ng/mL; 95% CI 0.00, 0.06, *P* = 0.043; *I*^2^ = 41.1% (with 12 studies)] in the severe group compared with the non-severe group. While no significant changes were observed in IL-6 [SMD = 0.54; 95% CI − 0.37, 1.45, *P* = 0.243; *I*^2^ = 95.5% (with 6 studies)] between the two groups (Additional file 2: Appendix 2d, Fig: A–E).

### Serum electrolytes

The pooled results of serum electrolytes among severe compared to non-severe patients indicated a significant decrease in the WMD of sodium [WMD = − 1.95 mmol/L; 95% CI − 2.87, − 1.03, *P* < 0.001; *I*^2^ = 75.5% (with 9 studies)], but non-significant difference on potassium [WMD = − 0.07 mmol/L; 95% CI − 0.18, 0.04, *P* = 0.206; *I*^2^ = 34.3% (with 9 studies)] (Additional file 2: Appendix 2e, Fig: A and B).

### Laboratory tests for coagulation functions

Pooled findings on laboratory tests for coagulation functions showed a significant increase in the WMD of fibrinogen [WMD = 0.80 g/L; 95% CI 0.32, 1.28, *P* = 0.001; *I*^2^ = 82.2% (with 4 studies)], PT [WMD = 0.63 s; 95% CI 0.27, 0.99, *P* = 0.001; *I*^2^ = 69.2% (with 8 studies)], and D-dimer [WMD = 0.18 mg/L; 95% CI 0.10, 0.27, *P* < 0.001; *I*^2^ = 99.3% (with 12 studies)] in severe vs. non-severe hospitalized patients (Additional file 2: Appendix 2f, Fig: A–C).

### Glucose level

We found a significant increase in glucose levels among the severe patients [WMD = 12.43 s; 95% CI 1.95, 22.91, *P* = 0.020; *I*^2^ = 0.0% (with 4 studies)] when compared with non-severe patients (Additional file 2: Appendix 2 g, Fig: A).

### Combined markers

The pooled findings on the new combined markers showed a significant increase in the SMD of NLR [SMD = 0.23; 95% CI 0.08, 0.37, *P *= 0.002; *I*^2^ = 14.6% (with 18 studies)] and a decrease in LCR [SMD = − 8.12; 95% CI − 10.05, − 6.18, *P* = 0.001; *I*2 = 98.6% (with 18 studies)], LeCR [SMD = − 1.47; 95% CI − 2.13, − 0.80, *P* = 0.001; *I*2 = 94.6% (with 17studies)], and (LeIR) [SMD = − 0.99; 95% CI − 1.98, − 0.00, *P* = 0.049; *I*2 = 93.6% (with 5 studies)] in severe vs. non-severe hospitalized patients infected by COVID-2019 (Additional file 2: Appendix 2 h, Fig: A–D).

Table 2 shows a summary of laboratory features in severe vs. non-severe hospitalized patients with COVID-19.

**Table 2:** Laboratory features in severe vs. non-severe hospitalized patients with COVID-19

Outcomes	Severe groups vs. non-severe groups		
	No	Pooled WMD (95% CI)	Heterogeneity (I2%, Pa)
Lymphocyte	22	− 0.43 (− 0.56, − 0.30)	90.1%, 0.000
Monocyte	10	− 0.06 (− 0.12, − 0.01)	71.8%, 0.000
Eosinophil	5	− 0.03 (− 0.05, − 0.00)	86.1%, 0.000
Hb	9	− 5.94 (− 8.23, − 3.64)	0.0%, 0.952
Platelet	17	− 27.97 (− 39.6, − 16.35)	55.8%, 0.003
Neutrophil	18	0.74 (0.16, 1.33)	74.6%, 0.000
WBC	21	0.55 (− 0.09, 1.19)	81.3%, 0.000
Albumin	12	− 4.20 (− 5.99, − 2.41)	73.9%, 0.000
ALT	15	6.65 (4.21, 9.09)	0.0%, 0.492
AST	14	11.91 (8.29, 15.53)	46.1%, 0.030
TBIL	9	0.08 (0.03, 0.14)	0.0%, 0.953
BUN	9	2.34 (0.66, 4.03)	39.3%, 0.106
Cr	14	0.08 (0.03, 0.12)	0.0%, 0.930
Creatine kinase	14	− 3.01 (− 12.91, 6.90)	51.7%, 0.013
Troponin I	5	0.27 (− 0.14, 0.67)	77.3%, 0.001
Myoglobin	3	8.11 (− 6.10, 22.33)	73.2%, 0.024
ESR	9	27.67 (22.94, 32.40)	22.3%, 0.245
CRP	18	36.61 (24.40, 48.82)	91.9%, 0.000
Lactate dehydrogenase	11	102.15 (72.76, 131.53)	50.3%, 0.028
Procalcitonin	12	0.03 (0.00, 0.06)	41.1%, 0.067
IL-6	6	0.54 (− 0.37, 1.45)	95.5%, 0.000
Sodium	9	− 1.95 (− 2.87, − 1.03)	75.5%, 0.000
Potassium	9	− 0.07 (− 0.18, 0.04)	34.3%, 0.144
Fibrinogen	4	0.8 (0.32, 1.28)	82.2%, 0.001
Protrombine	8	0.63 (0.27, 0.99)	69.2%, 0.002
D-dimer	12	0.18 (0.10, 0.27)	99.3%, 0.000
Glucose level	4	12.43 (1.95, 22.91)	0.0%, 0.433
Neutrophil‐to‐Lymphocyte ratio	18	0.23 (0.08, 0.37)	14.6%, 0.279
Lymphocyte-to-C-reactive protein ratio	18	− 8.12 (− 10.05, − 6.18)	98.6%, 0.000
Leukocyte-to-C-reactive protein ratio	17	− 1.47 (− 2.13, − 0.80)	94.6%, 0.000
Leukocyte-to-IL-6 ratio	5	− 0.99 (− 1.98, − 0.00)	93.6%, 0.000

HB, hemoglobin; WBC, white blood cell; ALT, alanine aminotransferase; AST, aspartate aminotransferase; TBIL, total bilirubin; BUN, blood urea nitrogen; Cr, Creatine; ESR, erythrocyte sedimentation rate; CRP, C-reactive protein; IL-6, interleukin 6

### Sensitivity analysis

We evaluated the effect of each study on the strength of the pooled WMDs or SMDs by excluding each study from the meta-analysis. We found no significant differences between the pre- and post-sensitivity pooled effect sizes for lymphocyte, hemoglobin, platelet, neutrophil, albumin, ALT, AST, TBIL, BUN, Cr, CK, troponin I, myoglobin, ESR, CRP, LDH, IL-6, sodium, fibrinogen, PT, D-dimer, glucose level, NLR, LCR, and LeCR. However after omitting Liu (Spngqiao) (a) et al. [20], the study on monocyte, (WMD = − 0.04, 95% CI − 0.10, 0.01), Liu (Spngqiao) et al. (b) [20] the study on eosinophil (WMD = − 0.02, 95% CI − 0.06, 0.01), Guan et al. [20], the study on WBC (WMD = 0.65, 95% CI 0.02, 1.27), Liu (Yanli) et al. [24], the study on PCT (WMD = 0.02, 95% CI − 0.004, 0.05), Huang et al. [7], the study on potassium (WMD = − 0.10, 95% CI − 0.18, − 0.02), and Deng et al. (b) [7], the study on LeIR (SMD = − 0.52, 95% CI − 1.35, 0.31), we found significant differences between pre- and post-sensitivity pooled effect sizes.

### Publication bias

The Egger’s regression and Begg’s rank correlation tests were performed to detect potential publication bias. These indicated no significant publication bias for lymphocyte, monocyte, eosinophil, hemoglobin, platelet, neutrophil, albumin, AST, ALT, TBIL, BUN, Cr, CK, troponin I, myoglobin, ESR, LDH, PCT, IL-6, potassium, fibrinogen, PT, D-dimer, glucose, NLR, LeCR, and LeIR. Because there was evidence of publication bias on WBC [Egger (*p* < 0.01), Begg (*P* = 0.13)], CRP [Egger (*p* < 0.01), Begg (*P* < 0.01)], sodium [Egger (*p* = 0.02), Begg (*P* = 0.29)], and LCR [Egger (*p* < 0.01), Begg (*P* < 0.01)], we conducted the non-parametric method (Duval and Tweedie) to estimate the findings of censored studies. There were no significant differences between before and after including censored studies for WBC, sodium, and LCR but not for CRP [before (WMD = 36.61 mg/L; 95% CI 24.40, 48.82) and after (WMD = 12.21 mg/L; 95% CI − 1.28, 25.84)].

## Discussion

To the best of authors’ knowledge, this is the first meta-analysis that elaborated on the differences between laboratory tests results of severe and non-severe confirmed cases of COVID-19. The results showed the significant decreased levels of lymphocyte, monocyte, eosinophil, hemoglobin, and platelet, while elevated neutrophil counts among the CBC indices in severe vs. non-severe patients. ALT, AST, TBIL, BUN, and Cr levels showed the significant increase, following a decrease in albumin level as the main liver and kidney outcomes in severe patients compared to non-severe ones. Inflammatory/infection markers (ESR, CRP, LDH, and PCT, but not IL-6), coagulation function tests (fibrinogen, PT, and D-dimer), and glucose were positively associated with the COVID-19 severity. However, serum sodium decreased in severe patients in comparison with non-severe group. Besides, potassium, and other cardiac-related factors were not associated with COVID-19 severity. It is worth to mention that combined ratios significantly decreased (LCR, LeCR, and LeIR6) and increased (NLR) in severe/critical compared to non-severe COVID-19 patients. The laboratory features presented by this meta-analysis could, to a large extent, be attributed to low oxygen saturation, respiratory failure, septic shock, and/or multiple organ dysfunction or failure compatible with the ARDS course of severe and critical COVID-19 types [2, 3].

COVID-19 is a systemic disorder affecting multiple organs. Abnormal kidney and liver function test results and elevated serum glucose might happen in more severe cases due to the following explanations. These include, but not limited to hypoxia, hypoperfusion as well as thrombosis caused by ARDS, shock and disseminated intravascular coagulation respectively. Aggravation of underlying disease in severe cases that are older and suffer from concomitant comorbidities also is another possibility for this feature. Drug-induced damages [23] to the liver are also one potential factor. Secondary hemophagocytic lymphohistiocytosis (HLH) characterized by hypercytokinemia with multi-organ failure [28] which occurred after viral infection in adults [29, 30] is another possible explanation for the stated laboratory features. Although one no peer-reviewed study by Zheng et al. [15] has demonstrated that organ damage in COVID-19 is mostly endorsed as organ damage caused by the virus itself rather than HLH. This claim needs more investigation and autopsy studies.

Myocardial damage in COVID-19, especially in severe cases, has been demonstrated previously [22, 24]; however, our analysis did not find significant higher level of myocardial enzymes in severe compared to non-severe cases. We believe more studies on the pathophysiology of such injury may help this issue to be clearer, one possibility could be interstitial infiltration of mononuclear cells instead of direct damage of myocardium in severe cases [23] which it is not in line with the finding of Zheng et al. [15].

The present findings showed a decreased in lymphocyte, eosinophil and monocyte counts but slightly increased of neutrophil in severe cases. Lymphopenia as a dysregulation in the immune response is mapped through more decrease in T cell and especially T helper cells in severe cases of COVID-19 [31]. The mechanism of this lymphopenia seems to be both due to the cytotoxic action of virus [6] and the collective characteristics of severe patients which are more likely to be older and have underlying diseases [24, 26, 32] which make them more susceptible to endothelial dysfunction and its correlated lymphopenia [33]. Findings of current study on decreased eosinophil and monocytes but increased neutrophils in severe cases need more investigations through future studies. These results highlight the potential of simple available tests besides the lymphocytes count for early screening in severe and critically ill COVID-19 cases.

The NLR was a commonly used index for determining the bacterial infections severity and for the prognosis of pneumonia and tumor patients [34, 35]. The current study suggests the increased NLR and decreased LCR of severe COVID-19 patients in which been recently reported [36] but to the best of our knowledge, our meta-analysis for the first time showed decrease in LeCR, and LeIR in severe COVID-19 patients compared to non-severe, indicating the poor prognosis of this pneumonia.

Lower hemoglobin level in more severe cases could be due to underlying medical conditions, malnutrition or coagulation abnormality. Coagulation abnormality is also contained in low platelet count, increased fibrinogen, D-dimer level and prolonged PT in severe patients.

An increase in PCT and neutrophil may be correlated with the concomitant bacterial infection in severe cases. Therefore, high PCT and neutrophil in two laboratory tests might be beneficial for the prognosis of severe cases. Despite that our meta-analysis found higher levels of ESR, CRP and LDH as inflammatory markers in severe cases of COVID-19, the results did not show increased IL-6 level in severe cases. This finding was mainly affected by the results of the study by Liu et al. [37] and should be confirmed with more investigations on IL-6 as well as other cytokines and chemokines.

Our findings provide evidence for the differentiation of severe cases of COVID-19 based on laboratory test results collected at the admission time. Our results also may provide the potential for efficient resource allocation in the era of scarcity of available resources including ICU.

However, inadequate evidence from outside China [27] may limit the generalizability of our results especially in terms of availability of the costly laboratory tests in low resource settings. Accordingly, as more data become available from outside of China in the ongoing weeks, it is highly recommended that new data are interpreted and compared with the present findings as the virus may differently affect based on both genetic and/or interplay environmental factors diversity. Moreover, heterogeneity of included studies as another limitation implies the need for further studies.

## Conclusions

In summary, our meta-analysis provides evidence for the differentiation of severe and non-severe cases of COVID-19 based on the laboratory test results on the time of admission. The results of CBC test, liver and kidney function tests, inflammatory/infection markers, serum electrolytes and glucose were significantly different between severe and non-severe cases of COVID-19. However, to confirm our results, we think further studies, particularly from other populations, are needed.

## Supplementary information

**Additional file 1.** Appendix 1 provides of search strategy from Scopus database.

**Additional file 2.** The mean difference forest plots of laboratory features in severe vs. non-severe hospitalized patients with COVID-19 are shown in Appendix 2: 2a–h.

## Data Availability

All data and materials can be accessed via RT and PN-S.
