# Supplementary material for: Laboratory features of severe vs. non-severe COVID-19 patients in Asian populations: a systematic review and meta-analysis
Source: Eur J Med Res. 2020 Aug 3;25:30. doi: 10.1186/s40001-020-00432-3 (PMC7396942; doi:10.1186/s40001-020-00432-3)
Supplement: Supplementary file 1 — Additional file 1. Appendix 1 provides of search strategy from Scopus database. [file 40001_2020_432_MOESM1_ESM.docx]

**Scopus 655**

( INDEXTERMS ( "2019 novel coronavirus infection" OR "COVID-19" OR "COVID19" OR "coronavirus disease 2019" OR "coronavirus disease-19" OR "2019-nCoV disease" OR "2019 novel coronavirus disease" OR "2019-nCoV infection" OR "2019-nCoV" OR "2019 novel coronavirus" OR "2019 coronavirus" OR "novel coronavirus" OR ( 2019 AND coronavirus ) ) OR TITLE-ABS-KEY ( "2019 novel coronavirus infection" OR "COVID-19" OR "COVID19" OR "coronavirus disease 2019" OR "coronavirus disease-19" OR "2019-nCoV disease" OR "2019 novel coronavirus disease" OR "2019-nCoV infection" OR "2019-nCoV" OR "2019 novel coronavirus" OR "2019 coronavirus" OR "novel coronavirus" OR ( 2019 AND coronavirus ) ) ) AND ( LIMIT-TO ( DOCTYPE , "ar" ) ) AND ( LIMIT-TO ( PUBYEAR , 2020 ) OR LIMIT-TO ( PUBYEAR , 2019 ) ) AND ( LIMIT-TO ( LANGUAGE , "English" ) )
