# Supplementary material for: Laboratory features of severe vs. non-severe COVID-19 patients in Asian populations: a systematic review and meta-analysis
Source: Eur J Med Res. 2020 Aug 3;25:30. doi: 10.1186/s40001-020-00432-3 (PMC7396942; doi:10.1186/s40001-020-00432-3)
Supplement: Supplementary file 2 — Additional file 2. The mean difference forest plots of laboratory features in severe vs. non-severe hospitalized patients with COVID-19 are shown in Appendix 2: 2a–h. [file 40001_2020_432_MOESM2_ESM.docx]

**Appendix 2a, Fig: A-G**. Complete blood cell count (CBC) with differential counts of white blood cells

**Fig. A**. Lymphocyte

**Fig. B**. Monocyte

**Fig. C**. Eosinophil

**Fig. D**. Haemoglubin (Hb)

**Fig. E**. Platelete

**Fig. F**. Neutrophil

**Fig. G**. WBC

**Appendix 2B, Fig: A-F**. Liver and kidney functions

**Fig. A**. Albumin

**Fig. B**. ALT

**Fig. C**. AST

**Fig. D**. TBil

**Fig. E**. BUN

**Fig. F**. Cr

**Appendix 2c, Fig: A-C**. Myocardial enzyme, Myoglobin

**Fig. A**. Creatine kinase (CK)

**Fig. B**. Troponin I (TnI)

**Fig. C**. Myoglobin

**Appendix 2d, Fig: A-E**. Inflammatory markers

**Fig. A**. ESR

**Fig. B**. CRP

**

**Fig. C**. Lactate dehydrogenase (LDH)

**Fig. D**. Procalcitonin (PCT)

**Fig. E**. IL-6

**Appendix 2e, Fig: A and B.** Serum electrolytes

**Fig. A.** Sodium (Na)

**Fig. B.** Potassium (K)

**Appendix 2f, Fig: A-C.** Coagulation functions

**Fig. A.** Fibrinogen

**Fig. B.** Protrombine (PT)

**Fig. C.** D-dimer

**Appendix 2g, Fig: A.** Glucose level

**Fig: A. Glucose level**

**Appendix 2h, Fig: A-D.** New markers of combinations

**Fig A.** Neutrophil‐to‐Lymphocyte ratio (NLR)

**Fig B.** Lymphocyte-to-C-reactive protein ratio (LCR)

**Fig C.** Leukocyte-to-C-reactive protein ratio (LeCR)

**Fig D.** Leukocyte-to-IL-6 ratio (LeIR)
